# Advances in Melanoma and Skin Cancers

**DOI:** 10.3390/ijms26051849

**Published:** 2025-02-21

**Authors:** Domenico Mallardo, Debora Basile, Maria Grazia Vitale

**Affiliations:** 1 Melanoma, Cancer Immunotherapy and Development Therapeutics Unit, Istituto Nazionale Tumori IRCCS “Fondazione G. Pascale”, Via Mariano Semmola, 53, 80131 Naples, Italy; dott.mariagrazia.vitale@gmail.com; 2 Unit of Medical Oncology, Ospedale San Giovanni di Dio, 88900 Crotone, Italy; deborabasile1090@gmail.com

Cutaneous melanoma, with a continuously rising incidence worldwide, represents one of the most aggressive forms of skin cancer. Despite accounting for only 4% of all skin cancers, it is responsible for 80% of skin cancer-related deaths, highlighting its particular aggressiveness and the critical need for effective therapeutic strategies. The global burden of melanoma has increased significantly over the past decades, with current estimates suggesting that over 331,722 new cases are diagnosed annually worldwide [1,2].

This rising incidence, coupled with the complexity of treating advanced disease, has driven intensive research efforts in developing more effective therapeutic approaches. Recent epidemiological studies have shown significant variations in melanoma incidence across different geographical regions and populations, with the highest rates observed in Australia and New Zealand.

Immunotherapy has revolutionized the treatment landscape of advanced melanoma over the past decade, marking a paradigm shift in cancer treatment. The introduction of immune checkpoint inhibitors (ICIs) has represented a particularly significant breakthrough, offering substantially improved survival compared to previous therapies.

This therapeutic revolution began with the understanding of how cancer cells evade immune surveillance and the subsequent development of strategies to reactivate the system’s natural ability to recognize and eliminate cancer cells.

The success of ICIs in melanoma has not only transformed patient outcomes but also paved the way for immunotherapy applications in other cancer types. Long-term survival data from pivotal clinical trials demonstrate the unprecedented durability of responses, with some patients maintaining disease control for several years [3,4,5].

However, a proportion of patients still experience poor outcomes after immunotherapy, as confirmed by several studies [6,7,8,9,10]. As a result, identifying predictive biomarkers of the response to immunotherapy is an important area of research, aiming to prevent overtreatment, unnecessary risk of toxicity, and wasteful resource usage. Since gene expression signatures in liquid biopsies are non-invasive and repeatable tests that may provide high specificity and sensitivity [11], they are widely investigated as potential predictive or prognostic markers in cancer patients, specifically in subjects with cutaneous melanoma [12,13,14].

The main goal of this Special Issue is to explore the most recent advancements in immunotherapy for patients with skin cancers. The studies included cover a wide range of research and treatment aspects, providing a comprehensive and in-depth view of the challenges and opportunities in this rapidly evolving field. This collection of studies provides a comprehensive and up-to-date overview of the main trends and future challenges in immunotherapy for skin cancers. Each study aims to better understand how we can continue to improve care for patients suffering with these devastating diseases.

A specific combined proteomic and transcriptomic signature was identified to predict the response to anti-PD-1 treatment in patients with metastatic melanoma. The researchers integrated whole proteome profiling of metastatic tissue with targeted transcriptomics to uncover the molecular signatures associated with treatment outcomes. The findings revealed that a combination of protein and gene signatures could predict poorer responses to immunotherapy. Patients with high expressions of these signatures exhibited significantly better progression-free (PFS) and overall survival (OS) compared to those with low expressions [15].

In another study, researchers investigated the anti-melanoma effects of miconazole, particularly its impact on mitochondrial functions. They treated human melanoma cell lines with miconazole and clotrimazole, observing the significant inhibition of cell proliferation and viability, along with reduced vascular mimicry. They found that miconazole decreased ATP levels and increased ROS production, indicating mitochondrial dysfunction. Additionally, miconazole altered the expression of several metabolites and genes involved in carnitine metabolism, which are crucial for mitochondrial function. These changes suggest that miconazole’s anti-melanoma effects are mediated through mitochondrial disruption and modulation of carnitine metabolism, ultimately leading to apoptosis in melanoma cells [16].

In one study, the effectiveness of combining Mcl-1 with Bcl-2/Bcl-xL/Bcl-w inhibitors was investigated in targeting melanoma cells. The researchers conducted in vitro experiments on melanoma cell lines with and without BRAF mutations. They used various inhibitors, including ABT-737, ABT-263, ABT-199, and S63845. The results showed that none were significantly effective when used alone. However, combining S63845 with each of the ABT inhibitors almost completely eliminated melanoma cell survival and induced apoptosis in 50% to 90% of the cells. This effect was associated with caspase activation, loss of mitochondrial membrane potential, phosphorylation of histone H2AX, and ROS production [17].

Moreover, the antitumor activity of GP-2250, a GAPDH inhibitor, on BRAF-mutated melanoma cell lines and benign melanocytes is examined. The results show that GP-2250 inhibits the proliferation and viability of melanoma cells in a dose-dependent manner without significantly affecting benign melanocytes. This indicates that GP-2250 could be a promising treatment for melanomas resistant to targeted therapies, highlighting the potential for new therapeutic strategies in combating this aggressive form of cancer [18].

The expression of programmed cell death ligand 1 (PD-L1) in cutaneous melanoma and its relationship with immunotherapy response is examined in another study. The authors discuss the controversies surrounding the clinical utility of PD-L1 assessment and suggest that specific evaluation methods could improve its predictive value for treatment outcomes. This research emphasizes the need for precise biomarkers to guide immunotherapy decisions and improve patient outcomes [19].

A comprehensive review of the status of molecular mechanisms of resistance to immunotherapy in oral malignant melanoma provides valuable insights into this challenging area. The authors analyze clinical studies on the use of immune checkpoint inhibitors and discuss future prospects for improving therapeutic efficacy and patient outcomes. This review highlights the ongoing efforts to overcome resistance and enhance the effectiveness of immunotherapy in treating oral malignant melanoma [20].

In a narrative review, the efficacy of pembrolizumab in advanced melanoma is examined. The authors review recent studies on the use of pembrolizumab, both as monotherapy and in combination with other treatments, and discuss potential new therapeutic options for patients who do not respond to immunotherapy. This review underscores the importance of ongoing research to expand the arsenal of effective treatments for advanced melanoma [21].

Furthermore, in a retrospective study, other researchers examined how pentoxifylline (PTX) and norcantharidin (NCTD) affect the expression of the p62 protein in melanoma cells, using both two-dimensional (2D) monolayer cultures and three-dimensional (3D) spheroids of B16F1 mouse melanoma cells. The researchers found that PTX decreases p62 expression in both 2D and 3D cultures, while NCTD increases p62 expression specifically in 3D cultures after 24 h. These findings suggest that PTX and NCTD could potentially regulate autophagy in melanoma cells, depending on disease stage. This regulation of autophagy could be crucial for developing new therapeutic strategies against melanoma [22].

Finally, advancements in immunotherapy have significantly transformed the therapeutic landscape for cutaneous melanoma, providing substantial clinical benefits. However, challenges remain, particularly in predicting patient responses and overcoming resistance. Ongoing research into biomarkers and novel therapeutic strategies is crucial for further improving clinical outcomes. The studies highlighted in this Special Issue underscore the importance of continuous innovation and interdisciplinary collaboration in melanoma research. These combined efforts pave the way for more personalized and effective treatments, ultimately aiming to reduce the global burden of this aggressive cancer.
